# Nrf2 Activation in Inflammatory Diseases: A Review of Natural and Synthetic Modulators

**DOI:** 10.1155/omcl/4538420

**Published:** 2026-03-10

**Authors:** Vitória B. Costa, Iolanda A. F. de Matos, Isabela R. G. Nogueira, Mariely A. de Godoi, Fábio R. M. Leite, Morgana R. Guimarães-Stabili

**Affiliations:** ^1^ Department of Diagnosis and Surgery, School of Dentistry at Araraquara, Sao Paulo State University (UNESP), Araraquara, 14.801-903, Brazil, unesp.br; ^2^ Section of Public Health, School of Dentistry, University of Utah, Salt Lake City, Utah, 84108, USA, utah.edu

**Keywords:** inflammation, inflammatory diseases, Nrf2, Nrf2 activators, therapeutic modulation

## Abstract

The nuclear factor erythroid 2–related factor 2 (Nrf2) pathway is a central regulator of the cellular antioxidant response, playing a key role in modulating inflammation and defending against oxidative stress‐induced damage. A range of natural and synthetic compounds, including dimethyl fumarate, bardoxolone, oltipraz, RTA‐408, ursodiol, curcumin, sulforaphane, and resveratrol, have been shown to activate Nrf2 via distinct molecular mechanisms, thereby enhancing the expression of cytoprotective and antioxidant genes. These mechanisms include direct dissociation from Keap1, activation of AMP‐activated protein kinase, and modulation of signaling pathways relevant to cellular stress and immune regulation. In inflammatory diseases such as rheumatoid arthritis, periodontitis, diabetes mellitus, and inflammatory bowel diseases, Nrf2 activation has been associated with attenuation of oxidative damage, suppression of proinflammatory mediators, and improved tissue homeostasis. However, sustained or dysregulated activation of Nrf2 may promote tumor cell survival, proliferation, and chemoresistance, particularly in oncologic contexts. This narrative review synthesizes mechanistic insights and preclinical and clinical evidence on the role of Nrf2 in inflammatory diseases and evaluates the therapeutic potential of its key activators. The dual nature of Nrf2, as both a cytoprotective and potentially oncogenic factor, highlights the importance of context‐specific and temporally controlled modulation. A better understanding of these dynamics is essential for optimizing clinical applications and minimizing therapeutic risks.

## 1. Introduction

Alternative treatments for inflammatory diseases rely on a deeper understanding of how signaling pathways can be modulated for therapeutic purposes.

To better understand the role of Nrf2, its mechanism of action, and its activators in inflammatory diseases such as periodontitis, a narrative literature review was conducted through a comprehensive search of the databases PubMed, Web of Science, and Google Scholar. Initially, a combination of the keywords “Nrf2 and mechanism of action” was used. Subsequently, keyword combinations specific to each selected inflammatory disease were employed, such as “Nrf2 and periodontitis.” Based on previously reviewed articles that identified different Nrf2 activators [1, 2], certain activators deemed most relevant in the literature were selected. Keyword combinations were then created for each chosen activator, for example, “Nrf2 and dimethyl fumarate.”

The inclusion criteria encompassed full‐text scientific articles published in international journals on the subject, published between 2009 and the present. Exclusion criteria included articles not written in English or those that did not present the correlations defined in the search strategy.

## 2. Nrf2 and Its Mechanism of Action

The transcription factor nuclear factor erythroid 2–related factor 2 (Nrf2) plays a fundamental role in regulating the antioxidant response, being a key component in the defense against oxidative stress. It is activated in response to several stimuli, including reactive oxygen species (ROS) and electrophilic agents. When activated, Nrf2 migrates to the cell nucleus, where it binds to antioxidant response elements (AREs) in the promoter regions of target genes, inducing the transcription of several antioxidant genes, including heme oxygenase‐1 (HO‐1) and glutathione S‐transferase (GST) [3]. This cascade of events culminates in a robust cytoprotective response that is essential for maintaining redox homeostasis and preventing cellular damage under stress.

**Figure 1:**
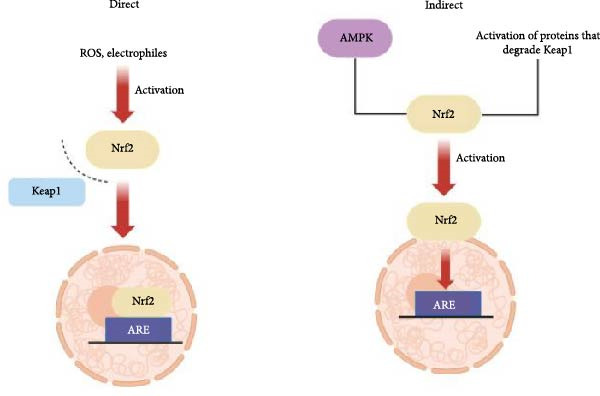
Direct and indirect mechanisms of Nrf2 activation. The most common form of activation occurs through the dissociation of Nrf2 from its inhibitor, Keap1, in response to stressful stimuli. As an indirect mechanism, the modulation of signaling pathways influences the dissociation of Nrf2 from Keap1. For example, the modulation of AMPK promotes Nrf2 activation. In addition, the activation of Nrf2 can occur through the activation of proteins that degrade Keap1, thereby enabling Nrf2 to have a lower stability.

Activation of Nrf2 can occur through several strategies, the most common being the dissociation of Nrf2 from its cytoplasmic inhibitor, Keap1 (Kelch‐like ECH‐associated protein 1). Under normal conditions, Keap1 promotes the degradation of Nrf2, maintaining its levels low. However, in response to stimuli, Keap1 undergoes modifications that disrupt its interaction with Nrf2, allowing Nrf2 to accumulate and translocate to the nucleus (Figure 1). This activation has been explored as a promising therapeutic strategy for a variety of inflammatory diseases, with several compounds being studied to promote Nrf2 activation in a controlled and effective manner [3].

Furthermore, indirect mechanisms of Nrf2 activation, involving the modulation of signaling pathways that influence the dissociation of Nrf2 from Keap1, are also described. Compounds such as resveratrol, for example, are known to activate Nrf2 indirectly by promoting the modulation of enzymes, including AMP‐activated kinase 1 (AMPK), which phosphorylates and activates Nrf2 (Figure 1) [4]. This process is crucial because it allows Nrf2 to exert its cytoprotective effects in cells under oxidative stress, as observed in the treatment of diabetic neuropathies [5]. Moreover, Nrf2 can also be activated through mechanisms that involve the degradation of Keap1 by specific proteins, which reduces Keap1‐mediated repression and thereby enhances Nrf2 stability and activity [6].

Beyond its canonical activation via Keap1 dissociation and nuclear translocation, Nrf2 interacts dynamically with other key regulators of cellular stress, including NF‐κB, MAPK, and PI3K/Akt signaling pathways. These interactions allow Nrf2 to exert immunomodulatory effects beyond oxidative defense, such as inhibition of inflammasome activation and modulation of metabolic reprogramming in immune cells. Furthermore, epigenetic regulation of Nrf2, via histone acetylation, microRNAs, and DNA methylation, has emerged as a crucial layer of control, especially in chronic inflammatory and oncogenic contexts. Understanding this regulatory crosstalk is vital for developing precise Nrf2‐targeted therapies that balance cytoprotection with oncogenic risk.

## 3. Biological Activities of Nrf2 in Inflammatory Diseases

**Figure 2:**
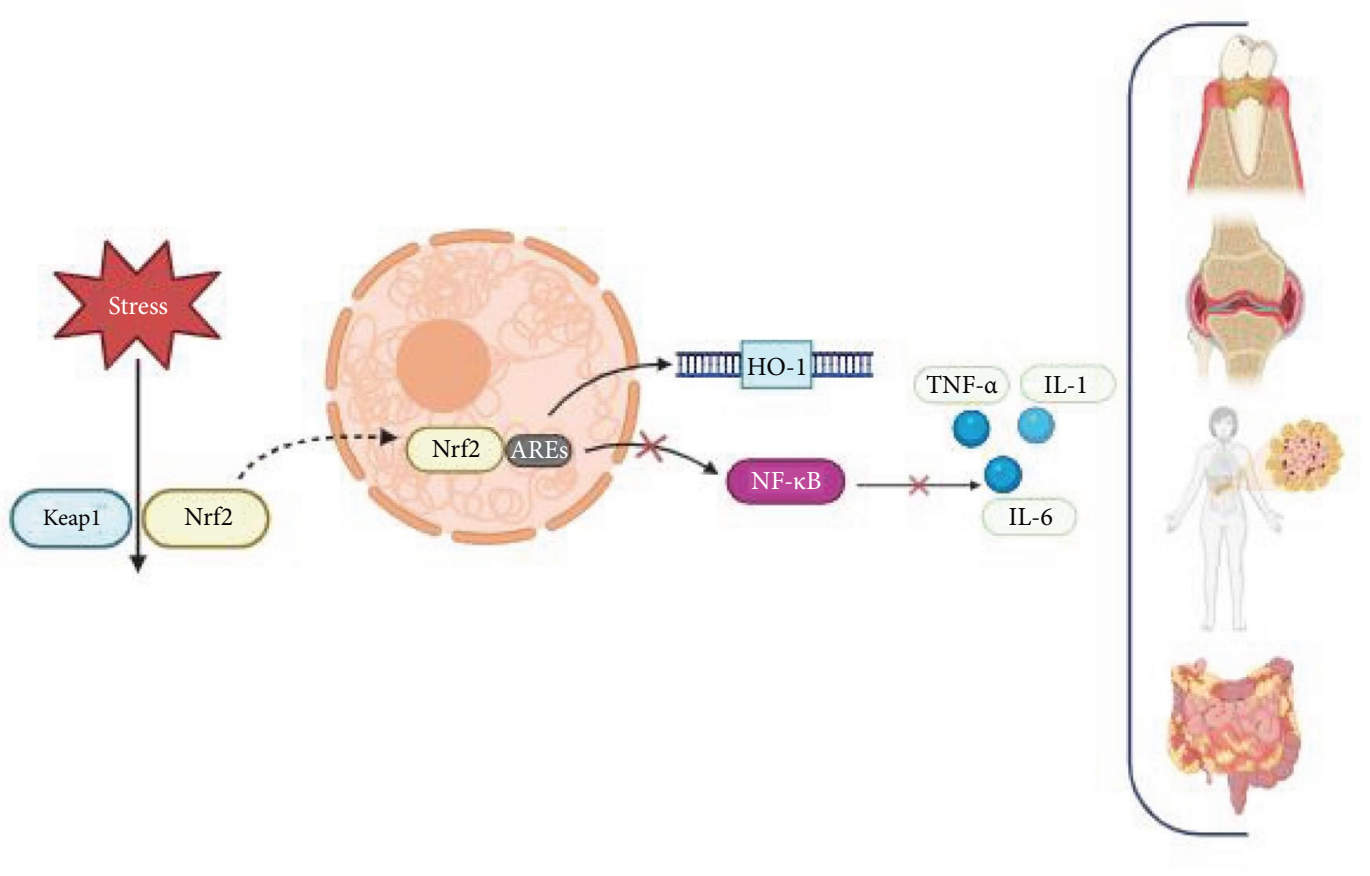
Mechanism of Nrf2 activation and its interference with the inflammatory response. Under basal conditions, Nrf2 is bound to a Keap‐1 molecule in the cell cytoplasm. When activated, Nrf2 migrates to the cell nucleus, where it binds to antioxidant response elements (AREs) in the promoter regions of target genes, inducing the transcription of a series of antioxidants. Nrf2 activation leads to inhibition of NF‐κB, decreasing the expression of proinflammatory cytokines that are involved in the pathogenesis of several inflammatory diseases.

Beyond its well‐established antioxidant role (Section 1), Nrf2 also modulates inflammation through crosstalk with pathways such as NF‐κB (nuclear factor kappa B) and MAPK (mitogen‐activated protein kinase), which are central regulators of inflammation, modulating the expression of several inflammatory mediators. This bidirectional crosstalk helps maintain a dynamic balance between inflammation and antioxidant defense, highlighting the complexity of cellular responses in inflammatory environments (Figure 2).

The state of Nrf2 activation in inflammatory diseases is of great importance in understanding the progression and severity of these conditions (Table 1). In chronic inflammatory diseases such as ulcerative colitis and rheumatoid arthritis, Nrf2 activation is often compromised, contributing to increased oxidative stress and inflammation [14, 18]. In metabolic diseases such as diabetes mellitus, the inactivation of the Nrf2 pathway has been linked to inflammatory complications such as diabetic neuropathy and periodontitis [5, 6]. Studies have demonstrated that Nrf2 activation can attenuate inflammation and oxidative damage associated with these complications, suggesting that modulation of the pathway may have significant therapeutic potential in managing the inflammatory complications of diabetes [5, 6].

**Table 1:** Biological activities of Nrf2.

Inflammatory diseases	Role of Nrf2	Author and year
Periodontitis	Reduction of inflammation, oxidative stress, bone alveolar resorption and symtoms associated wih the diasease	Li et al. [7, 8]; Zhang et al. [9]
Rheumatoid arthritis	Decreased ROS levels, attenuation of inflammation, cytoprotection against drug toxicity in the kidneys and liver, possible decreased efficacy of antirheumatic drugs and increased survival of pathological cells	Manda et al. [10]
Cancer	Elevated Nrf2 levels are associated with tumor progression and poor prognosis. Induction of Nrf2 before disease could prevent carcinogenesis	Lan et al. [11]
Diabetes	Individuals with diabetes mellitus showed decreased levels of Nrf2. Nrf2 activation promotes ROS reduction and oxidative stress‐related apoptosis	Sireesh et al. [12]; Zhang et al. [5]
Inflammatory bowel diseases	Patients with colitis have increased oxidative mediators and reduced Nrf2 expression. Nrf2 activation alleviates disease symptoms through activation of antioxidant components and inhibition of pro‐inflammatory components	Zhu et al. [13]; Peng et al. [14]
Obesity	Nrf2 activation promotes reduction of inflammation and oxidative stress caused by the disease	Song et al. [15]
Hypertension	During disease, there is a negative regulation of Nrf2, leading to disruption of homeostasis. Activation of the pathway is associated with reduced oxidative stress and attenuation of inflammation	Wafi [16]; Fu et al. [17]

However, while the therapeutic regulation of Nrf2 presents a promising approach for treating various chronic inflammatory diseases, it is crucial to weigh the benefits and drawbacks of its continuous activation [10, 19], as excessive activation may lead to adverse effects, including increased resistance to chemotherapy drugs in cancer cells. In the tumorigenesis process, Nrf2 appears to play a role in the biosynthesis of oncoproteins, the elimination of ROS through the activation of antioxidant genes, and the removal of toxic carcinogens, ultimately promoting tumor cell survival and proliferation [11, 20, 21]. In addition, evidence suggests that sustained Nrf2 activation in chronic inflammatory diseases such as rheumatoid arthritis may favor the persistence of pathogenic cells and reduce therapeutic efficacy [19]. Therefore, strategies aimed at balancing Nrf2 activation, to maximize its antioxidant and anti‐inflammatory effects while minimizing associated risks, represent a highly relevant area of study for the development of novel therapies for inflammatory diseases [11, 21] (Table 1).

### 3.1. Rheumatoid Arthritis

Rheumatoid arthritis (RA) is a chronic inflammatory disease that primarily affects the joints, resulting in pain, swelling, and ultimately, joint tissue destruction. A hallmark of RA is the imbalance between prooxidant and antioxidant mechanisms, resulting in increased oxidative stress. Nrf2 plays a central role in cellular defence against oxidative stress, emerging as a key regulatory factor in this context. Nrf2 modulates RA‐associated inflammation via antioxidant and detoxifying responses already described (Section 1), contributing to redox balance and immune regulation [22].

During disease progression, Nrf2 function may become compromised, resulting in an insufficient antioxidant response and increased inflammation. Research suggests that inadequate Nrf2 activation in RA patients is associated with increased disease severity [22]. The failure to properly activate antioxidant genes leads to increased cellular and tissue damage within affected joints [23]. Furthermore, reduced expression of Nrf2 and its target genes has been observed in the inflamed tissues of RA patients, indicating an impairment of this crucial defense pathway [24].

Activation of Nrf2 in experimental RA models has shown promising results, leading to significant symptom improvement, including reduced joint inflammation and protection against joint damage [25], which suggests potential translational relevance and warrants further clinical investigation. These benefits are attributed to the negative modulation of proinflammatory cytokine production [26].

However, the use of Nrf2 as an adjuvant therapy in RA remains controversial. While pathway activation can help reduce inflammation and oxidative stress, concerns exist that excessive activation may lead to adverse effects, such as promoting the survival of pathogenic cells [10]. Therefore, precise modulation of the Nrf2 pathway is crucial to maximizing therapeutic benefits while minimizing unwanted side effects.

Other studies suggest that Nrf2 activation may provide additional benefits in osteolytic inflammatory diseases such as RA by suppressing osteoclastogenesis, an essential process in the bone destruction observed in RA. By inhibiting the expression of interleukin‐6 (IL‐6), a key inflammatory cytokine, Nrf2 activation may help protect against disease‐associated bone loss [27]. In macrophages, Nrf2 activation has been shown to attenuate osteoclast differentiation and osteolytic activity while suppressing genes related to bone resorption [28].

Furthermore, Nrf2 inhibition has been found to exacerbate disease symptoms, as suppression of this pathway increases the proliferation and invasion of fibroblast‐like synoviocytes [29], which are involved in joint destruction in RA. This finding reinforces the crucial role of Nrf2 in limiting inflammatory and destructive processes in RA.

In summary, Nrf2 activation holds significant therapeutic potential for rheumatoid arthritis, particularly in controlling inflammation and preventing bone destruction. However, precise modulation of this pathway is essential to avoid adverse effects, underscoring the complexity of targeting Nrf2 for therapeutic purposes [19].

### 3.2. Periodontitis

Periodontitis is a chronic inflammatory disease that affects the supporting tissues of the teeth, leading to alveolar bone destruction and, in severe cases, tooth loss [30]. It is not merely a simple inflammatory response, but rather a complex network of cellular interactions. Gingival fibroblasts, for instance, can interact with osteoclasts to promote alveolar bone resorption, while neutrophils and macrophages modulate the immune response through the release of cytokines and chemokines. Furthermore, the identification of distinct cellular subpopulations, such as fibroblasts exhibiting immunomodulatory or tissue‐remodeling phenotypes, highlights the intricate nature of host responses associated with the disease [31].

In human gingival fibroblasts (HGFs), Nrf2 contributes to inflammatory regulation, complementing its canonical antioxidant function previously discussed [32]. In cells stimulated by lipopolysaccharide (LPS) from *Porphyromonas gingivalis*, the activation of Nrf2 resulted in a significant reduction in the release of inflammatory mediators, including IL‐6, IL‐8, PGE2, and nitric oxide (NO) [32].

Regarding neutrophils, Nrf2 activation promotes the induction of antioxidant genes, such as Nqo1, Hmox‐1, and Cat, which significantly reduces the production of ROS during the oxidative burst [33]. Furthermore, Nrf2 attenuates the expression of proinflammatory genes such as Tnfα, Ccl3, and Cxcl2, thereby limiting the inflammatory response [33]. In terms of migration, Nrf2‐deficient neutrophils exhibited reduced spontaneous migration as well as diminished chemotaxis. This suggests that Nrf2 is essential for the sensitivity and efficient motility of neutrophils in response to chemokines. Thus, Nrf2 selectively regulates key neutrophil functions, including ROS production, inflammatory cytokine expression, and migration, contributing to the control of inflammation and the maintenance of tissue homeostasis [33].

In macrophages, Nrf2 can directly repress the transcription of pro‐inflammatory genes such as IL‐6 and IL‐1β in LPS‐activated cells, independently of its antioxidant function. This occurs through interference with RNA polymerase II recruitment, thereby inhibiting the transcription of these inflammatory genes [34]. Furthermore, studies indicate that Nrf2 is essential for macrophage polarization toward the anti‐inflammatory M2 phenotype, particularly in tumor microenvironments, where metabolites such as lactate can activate Nrf2 and promote a regulatory immune response [35]. Finally, Nrf2 activation in macrophages is associated with metabolic reprogramming, facilitating mitochondrial adaptations and fine‐tuning the innate immune response, which is crucial for the effective function of these cells during infections and inflammation [34, 35].

In CD4^+^ T lymphocytes, Nrf2 activation promotes polarization toward the Th2 phenotype, characterized by the production of cytokines such as IL‐4 and IL‐13, while suppressing the Th1 profile, which is associated with IFN‐γ production [36]. This mechanism is particularly relevant in allergic and autoimmune contexts, where modulation of the Th1/Th2 balance is critical. In B lymphocytes, although less extensively studied, evidence suggests that Nrf2 also influences their function by modulating chronic inflammatory responses and affecting antibody production. Nrf2 activation may regulate B cell responses to inflammatory stimuli, although the specific mechanisms involved require further investigation [37].

The periodontal environment is complex, involving diverse cell types that interact through Nrf2‐related pathways. Recent studies have explored the role of Nrf2 in modulating the inflammatory response and oxidative stress in periodontitis, suggesting that activation of this pathway may have significant therapeutic potential [38]. The protective role of Nrf2 in periodontitis has been investigated in different experimental contexts [9]. A study evaluating the effect of Biochanin A, an isoflavone, demonstrated that this compound alleviated gingival inflammation and alveolar bone loss in rats with experimental periodontitis. This effect was associated with the activation of the Nrf2 pathway, which reduced the production of proinflammatory cytokines and oxidative stress, suggesting that modulation of the pathway may be an effective strategy to protect periodontal tissues [9].

Data from a study evaluating the relationship between diabetes mellitus and periodontitis revealed that diabetes can exacerbate periodontitis by intensifying oxidative damage and downregulating Nrf2 [6]. Reduced Nrf2 activity in diabetic individuals contributed to increased oxidative stress and inflammation, worsening the destructive effects of periodontitis. These findings suggest that Nrf2 activation may be particularly important in patients with comorbidities such as diabetes, although clinical evidence remains limited and further studies are needed to determine whether systemic Nrf2 modulation can produce consistent therapeutic outcomes in humans [6].

Furthermore, the Nrf2 pathway has been implicated in studies investigating the macrophage response in periodontitis. Research indicates that human adipose tissue‐derived stem cells may exert therapeutic effects on the disease by promoting M1‐to‐M2 macrophage polarization, facilitating tissue repair, and reducing bone loss in rats with induced periodontitis [39]. Activation of the Nrf2 pathway in these models led to a decreased inflammatory response and enhanced protection against bone destruction, highlighting its central role in immune regulation in periodontitis [39].

In preclinical models, Nrf2 activation has shown protective effects in periodontitis. For example, silibinin has been shown to reduce inflammation and oxidative stress in animal studies, indicating its potential for future therapeutic strategies, pending clinical evaluation. This study reinforces the idea that natural compounds can be explored as potential adjuvant therapies in periodontitis, modulating Nrf2 to protect periodontal tissues [8].

Additionally, the effects of Nrf2 were investigated in human gingival fibroblasts stimulated by advanced glycation end products (AGEs), a common condition in diabetic patients. In vitro studies suggest that Vitamin D may exert anti‐inflammatory effects via Nrf2 pathway activation in gingival fibroblasts; however, the in vivo relevance remains to be confirmed [40]. When activated, Nrf2 reduced the production of proinflammatory cytokines such as IL‐6 and IL‐8 and counteracted the harmful effects of AGEs, contributing to tissue protection and delaying premature aging [40].

Finally, a systematic review and preclinical meta‐analysis demonstrated the protective role of Nrf2 in periodontitis, confirming that Nrf2 activation inhibits oxidative stress and inflammation, thereby preventing disease progression [7]. These findings, based on animal model studies (murine) with systemic administration of various Nrf2 activators, such as resveratrol, suggest that targeting Nrf2 activation could offer an effective therapeutic approach for periodontitis, preserving tooth‐supporting tissues and promoting periodontal health [7].

### 3.3. Diabetes

Diabetes mellitus is a metabolic condition marked by chronically elevated blood glucose levels, stemming from deficiencies in insulin secretion, the body’s responsiveness to insulin, or both. Two major forms are recognized: type 1 diabetes, an autoimmune disorder in which pancreatic beta cells are destroyed, leading to complete insulin deficiency; and type 2 diabetes, where insulin resistance precedes a progressive decline in insulin production. Sustained hyperglycemia is associated with a wide range of complications, including damage to the cardiovascular, nervous, renal, and visual systems.

The transcription factor Nrf2 contributes significantly to metabolic regulation in diabetes by modulating several protective pathways [41]. Its activation enhances the expression of antioxidant enzymes—such as heme oxygenase‐1 (HO‐1) and superoxide dismutase (SOD)—which act to neutralize ROS and limit oxidative damage. Additionally, Nrf2 supports pancreatic beta‐cell integrity and function, thereby contributing to insulin secretion and improved glycemic regulation. It also plays a role in lipid metabolism, promoting reductions in circulating triglycerides and cholesterol levels. Moreover, Nrf2 influences cellular glucose metabolism by regulating genes associated with glycolysis and mitochondrial energy production, leading to more efficient glucose utilization and decreased blood glucose concentrations [42].

Clinical studies involving patients with type 2 diabetes have demonstrated an inverse relationship between Nrf2 levels and inflammatory cytokine concentrations, implying that insufficient Nrf2 expression may exacerbate early inflammatory processes and contribute to disease advancement [12].

The relationship between diabetes and periodontitis has also been explored in the context of Nrf2 regulation. Reduced expression of Nrf2 in diabetic conditions has been linked to increased oxidative injury and enhanced periodontal destruction, suggesting that Nrf2 deficiency may worsen inflammatory responses in comorbid cases [6]. Experimental data further show that Nrf2 activation can suppress ferroptosis, attenuate myocardial fibrosis, and improve cardiac recovery following infarction in diabetic animal models [43]. These findings underscore the multifaceted regulatory functions of Nrf2 in preventing diabetes‐associated tissue damage, although clinical translation remains to be fully established.

The therapeutic potential of Nrf2 is further supported by research on bioactive compounds. Resveratrol, a naturally occurring polyphenol found in grapes, has demonstrated the ability to alleviate diabetic neuropathy by activating Nrf2 signaling, resulting in anti‐inflammatory and antioxidant effects [5]. This evidence suggests that targeting the Nrf2 pathway may offer promising strategies for managing oxidative stress and inflammation in diabetic patients.

The importance of Nrf2 is also highlighted in studies investigating the use of natural compounds as potential therapies for diabetes and their complications. Resveratrol, a polyphenol found in grapes, can attenuate diabetic neuropathy by activating the Nrf2 pathway, leading to significant anti‐inflammatory effects [5]. These findings suggest that Nrf2 activation may be an effective strategy for managing oxidative stress and inflammation associated with the disease, bringing potential therapeutic benefits to patients.

### 3.4. Inflammatory Bowel Diseases

Inflammatory bowel diseases (IBD) are a group of chronic conditions affecting the gastrointestinal tract, the most common of which are Crohn’s disease and ulcerative colitis. These diseases are characterized by persistent inflammation of the intestine, which can lead to symptoms such as abdominal pain, diarrhea, fatigue, and weight loss. Although the exact origin of IBD is not fully understood, it is believed to involve a combination of genetic, environmental, and immunological factors, resulting in a dysregulated immune response in the intestine [18].

A central component in the pathogenesis of IBD is oxidative stress, which occurs when there is an imbalance between the production of free radicals and the body’s ability to neutralize them. In this condition, Nrf2 plays a critical role as a transcription factor regulating the expression of antioxidants and cytoprotective genes, thus contributing to cellular defense. In IBs, the regulation of the Nrf2 pathway may be impaired, exacerbating inflammation and tissue damage. Therefore, several studies have investigated the role of Nrf2 in experimental models of IBD [18].

Activation of Nrf2 provides beneficial effects in controlling the disease through several mechanisms, such as promoting the expression of antioxidant genes [44], (e.g., superoxide dismutase, catalase and glutathione peroxidase), which neutralize increased expression of ROS; inhibition of proinflammatory factors such as TNF‐α, IL‐1β, IL‐6 and COX‐2 through inhibition of nucleat factor‐kapp beta (NF‐κB); improvement of the integrity of the intestinal epithelial barrier through the expression of proteins known as “tight junctions” such as claudin and occludin, capable of reducing intestinal permeability, thus protecting against bacterial translocation. Furthermore, Nrf2 modulates autophagy (the process by which cells remove damaged organelles) by activating genes that help control chronic inflammation and reduce damage to intestinal cells [44, 45].

The modulation of Nrf2 by bioactive compounds, such as dieckol and carbocisteine, in models of colitis induced by sodium dextran sulfate (DSS) in animal models and cell studies, has been shown to reduce inflammation by suppressing NF‐κB activity, thereby reducing the expression of proinflammatory cytokines, including TNF‐α and IL‐1β [13]. In addition, Nrf2 regulates the HO‐1 pathway and suppresses the expression of COX‐2 and inducible nitric oxide synthase (iNOS), which are responsible for amplifying the inflammatory response. In addition to reducing inflammation, Nrf2 activation has been associated with reduced oxidative stress through the induction of antioxidant enzymes (SOD, catalase and glutathione peroxidase). There is also regulation of the expression of NAD(P)H quinone oxidoreductase‐1 (NQO1), which plays a role in the detoxification of quinones; and reduction of the production of reactive nitrogen species, which can cause oxidative damage to DNA and other biomolecules [13, 46].

The metabolic regulation of Nrf2 constitutes a central axis linking the gut, the immune system, and systemic oxidative control, supported by interactions among dietary compounds, microbial metabolites, and cellular antioxidant pathways. Sulforaphane and short‐chain fatty acids (SCFAs) exemplify this integration by promoting Nrf2 activation, inhibiting NF‐κB, and strengthening epithelial and endothelial barriers [47, 48]. This dynamic is reinforced by recent findings on deer oil [49] and the flavonoid Carthamin yellow [50], which restore the intestinal microbiota, increase SCFA production, and activate the Nrf2/HO‐1 and Nrf2/GPX4 pathways, thereby attenuating inflammation and ferroptosis. Complementarily, authors demonstrated that the metabolite indole‐3‐lactic acid (ILA), produced by *Bifidobacterium longum* subsp. *infantis* when cultured in human milk oligosaccharides, exerts potent anti‐inflammatory effects through coordinated activation of AhR and Nrf2 receptors in intestinal epithelial cells. ILA suppresses IL‐8 expression and activates antioxidant genes such as *GPX2*, *SOD2*, and *NQO1*, modulating immune responses and intestinal redox balance [51]. Thus, microbial and metabolic control of Nrf2 emerges as a multifactorial physiological nexus in which microbiota‐derived metabolites, such as SCFAs and ILA, together with dietary Nrf2‐activating compounds, sustain gut–immune communication, preserve oxidative homeostasis, and prevent systemic inflammation.

Corroborating these studies, several scientific investigations have highlighted the activation of Nrf2 by compounds of natural origin and their bioactives as one of the main mechanisms underlying the anti‐inflammatory effects observed in experimental models of colitis in rodents [52], suggesting that modulation of this signaling pathway may constitute a promising therapeutic strategy to protect intestinal tissues from damage caused by prolonged inflammation [52, 53].

The importance of Nrf2 in protecting against oxidative stress and regulating the inflammatory response in the intestine is emphasized in these studies, showing that activation of the pathway represents a promising strategy for the development of new therapies for IBD, potentially reducing the need for more aggressive treatments and that activation of the pathway represents a promising strategy for developing new therapies for IBD, potentially reducing the need for more aggressive treatments and their associated side effects. Thus, interventions that aim to modulate Nrf2 may play a crucial role in the management and treatment of IBD.

### 3.5. Oral Cancer

Oral cancer encompasses malignant tumors affecting structures such as the tongue, gums, palate, and oral cavity lining, with squamous cell carcinoma (SCC) representing the predominant histological subtype [54]. Often diagnosed at advanced stages due to nonspecific early symptoms, treatment typically involves surgical resection, chemotherapy, and radiotherapy. Despite these approaches, therapeutic resistance and high recurrence rates remain significant challenges in clinical management.

The transcription factor Nrf2, widely recognized for its role in redox balance, has been implicated in both tumor suppression and cancer progression [11]. Under physiological conditions, Nrf2 activation promotes cellular defense against oxidative stress, a key factor in carcinogenesis. However, in oral malignancies, sustained or aberrant Nrf2 activation—often due to mutations in its negative regulator, KEAP1—can enhance tumor cell survival and resistance to treatment. One study found that advanced glycation end products (AGEs) modulate Nrf2 and Bcl‐xl expression in oral cancer cells, potentially contributing to impaired apoptotic responses and increased oxidative resistance, thereby supporting malignant progression [55].

Cisplatin, a cornerstone of chemotherapy for oral SCC, is particularly susceptible to resistance mechanisms involving the Nrf2‐KEAP1 axis [56]. Tumor cells harboring KEAP1 mutations may exhibit constitutive Nrf2 activation, resulting in the upregulation of genes involved in ROS neutralization and drug efflux. This reduces cisplatin‐induced DNA damage, diminishing its cytotoxic effect. Experimental data suggest that targeting this pathway, by silencing Nrf2 or restoring wild‐type KEAP1, can resensitize tumor cells to cisplatin, thereby limiting tumor growth and metastasis. Consequently, the Nrf2‐KEAP1 interaction and its downstream pathways have emerged as potential therapeutic targets in drug‐resistant oral cancers [56].

Interest has also grown in the use of pharmacological inhibitors to suppress Nrf2 activity in tumor contexts. Among them, brusatol and ML385 are two of the most widely studied compounds. Brusatol, although initially described as an Nrf2 inhibitor that promotes its degradation, lacks selectivity and may exert cytotoxic effects on nonmalignant cells [57, 58]. ML385, on the other hand, exhibits greater specificity by binding to the Neh1 domain of Nrf2 and blocking its transcriptional activity. Preclinical models have demonstrated that ML385 can counteract chemoresistance in tumors with KEAP1 deficiency. Furthermore, combinatory regimens using agents like metformin or sorafenib have been shown to induce ferroptosis through modulation of the p62‐KEAP1‐Nrf2 pathway, suggesting novel directions for cancer therapy [59].

### 3.6. Hypertension

Hypertension is a prevalent chronic disorder characterized by persistently elevated blood pressure and is one of the leading risk factors for cardiovascular complications such as stroke and myocardial infarction [60]. Often referred to as a “silent disease,” it typically progresses without noticeable symptoms during its early stages, which hampers timely diagnosis and preventive care. The underlying mechanisms driving hypertension are complex and multifactorial, with inflammation and oxidative stress recognized as major contributors to its pathophysiology [60].

Sustained inflammation plays a pivotal role in the development of vascular dysfunction associated with hypertension. Evidence suggests that proinflammatory cytokines and excessive ROS production disrupt endothelial integrity, thereby elevating vascular resistance and contributing to increased blood pressure. This prooxidant and inflammatory environment promotes a cycle of endothelial damage and impaired vascular homeostasis, reinforcing hypertensive pathology [61].

The Nrf2 signaling pathway has garnered attention for its role in modulating oxidative and inflammatory responses in hypertension. In experimental models, Nrf2 activation has been associated with improved cardiovascular parameters and attenuated inflammatory profiles, pointing to a protective function in blood pressure regulation [16]. Mechanistically, Nrf2 promotes the transcription of antioxidant enzymes such as HO‐1 and NAD(P)H quinone oxidoreductase, while concurrently inhibiting NF‐κB signaling, thereby downregulating inflammatory mediators such as TNF‐α and IL‐6. Moreover, Nrf2 activity influences autonomic regulation by reducing excessive sympathetic output, a known driver of elevated blood pressure. The protective role of Nrf2 also extends to vascular tissues, where its activation enhances endothelial resilience against oxidative insults, restoring vascular function and limiting inflammatory infiltration [16].

Pharmacological agents commonly used to treat hypertension may exert part of their effects through Nrf2 modulation. For instance, verapamil, a widely used calcium channel blocker, has been shown to activate Nrf2 by promoting the autophagic degradation of Keap1, the repressor of Nrf2 [62]. This activation leads to enhanced antioxidant defense and a reduction in inflammatory signaling, suggesting an added therapeutic benefit beyond calcium channel inhibition.

Nrf2 activity within central autonomic regulatory centers has also been implicated in blood pressure control. In models of neurogenic hypertension, activation of Nrf2 in the rostral ventrolateral medulla reduces oxidative stress and lowers systemic arterial pressure [63]. Similarly, central administration of AICAR, an AMPK pathway activator, induces Nrf2 expression in the hypothalamic paraventricular nucleus, leading to a marked antihypertensive effect in experimental animals [17].

In summary, hypertension involves a convergence of inflammatory and oxidative mechanisms that disrupt cardiovascular regulation. The Nrf2 pathway emerges as a central modulator of these processes, with evidence supporting its therapeutic potential through both peripheral and central mechanisms. Targeting Nrf2 activation could represent an innovative strategy to complement current antihypertensive therapies.

### 3.7. Obesity

Obesity is a chronic metabolic disorder defined by an excessive accumulation of adipose tissue, and it is widely associated with increased risk for type 2 diabetes, cardiovascular disease, and several types of cancer. In recent years, the link between obesity and systemic inflammation has been emphasized, particularly the recognition that adipose tissue in obese individuals sustains a state of chronic, low‐grade inflammation [64]. This inflammatory milieu contributes significantly to metabolic disturbances and the onset of comorbid conditions. Among the molecular regulators involved, Nrf2 has emerged as a key transcription factor responsible for orchestrating antioxidant and anti‐inflammatory gene expression [64].

Preclinical studies have demonstrated that activating the Nrf2 pathway may counteract some of the deleterious effects of obesity, particularly those induced by high‐fat diets. For example, treatment with 1‐methylnicotinamide (1‐MNA), a derivative of niacin, was shown to enhance Nrf2 expression while inhibiting NF‐κB activation, thereby reducing both oxidative stress and inflammation [15]. These results suggest that modulation of Nrf2 signaling could be protective against obesity‐induced cardiovascular complications.

Corroborating these findings, another study examining the impact of Nrf2 in models of obesity‐related cardiac dysfunction reported that Nrf2 activation not only improved cardiac function but also attenuated systemic inflammatory responses in animals that were fed a high‐fat diet [21]. These data reinforce the view that Nrf2 plays a protective role in limiting obesity‐induced cardiovascular injury. Moreover, recent evidence has revealed that stimulation of the TRPV3 receptor activates the NRF2/FSP1 axis, promoting lipolytic activity and reducing adipose tissue accumulation, alongside anti‐inflammatory effects [65].

The influence of weight‐loss interventions on Nrf2 activity has also been evaluated. Findings from a study on bariatric surgery demonstrated that the procedure significantly enhanced Nrf2 expression while concurrently reducing systemic inflammation [66]. These results suggest that part of the anti‐inflammatory benefit observed after surgical weight reduction may involve Nrf2‐dependent mechanisms.

Collectively, findings from animal models highlight the central role of Nrf2 in mitigating inflammation and metabolic disturbances associated with obesity. These insights position Nrf2 as a promising therapeutic target for obesity‐related conditions, although further clinical studies are needed to validate these effects in humans.

Building upon these findings, numerous Nrf2‐activating agents have been identified in the literature. In the following sections, we explore specific compounds with documented activity on this pathway and their potential applications in the treatment of inflammatory diseases (Tables 2–4).

**Table 2:** Pharmacological activators of Nrf2—Clinical trials.

Nrf2 activator	Study (author and year)	Study design	Principal findings	Adverse events
Dimethyl fumarate	Gold [67]	Phase III (Final ENDORSE)	DMF has a favorable benefit–risk profile, with sustained efficacy and well‐characterized safety over 10 years of treatment for multiple sclerosis	Most AEs were mild to moderate, and the incidence did not increase over time
	Sator [68]	Phase III	Effective and safe in the treatment of severe plaque psoriasis in patients in Austria	No serious adverse reaction

Bardoxolone	Lewis [69]	Phase III	Bardoxolone methyl increased estimated glomerular filtration rate (eGFR), indicating an improvement in renal function in patients with type 2 diabetes mellitus and stage 4 chronic kidney disease	Increases in serum concentrations of alanine aminotransferase (ALT), aspartate aminotransferase (AST), and gamma‐glutamyl transferase (GGT)
	Rossing [70]	Phase III	Increased estimated glomerular filtration rate (eGFR) and urinary albumin‐creatinine ratio (UACR) in patients with type 2 diabetes mellitus and stage 4 kidney disease	Significant increase in heart failure events

Oltipraz	Kim [71]	Phase II	Significant reduction in hepatic fat content in the groups receiving oltipraz, in a dose‐dependent manner in patients with nonalcoholic fatty liver disease	Gastrointestinal symptoms or disturbances were encountered more frequently; however, the incidence of adverse drug reactions was comparable among the three treatment groups
	Kim [72]	Phase II	No significant differences in the improvement of liver fibrosis between the oltipraz‐treated groups and the placebo group, although there were some trends toward improvement in specific markers in the 60 mg group	No serious toxicities or significant adverse effects were observed during the study period

Omaveloxolone	Creelan, 2017 [73]	Phase I	Decrease in the expression of nitration markers (NT) and the inducible isoform of nitric oxide synthase (iNOS) in treated tumor biopsies, indicating a possible reduction in tumor inflammation	Most are mild to moderate in severity, with a predominance of common symptoms and laboratory abnormalities that are monitorable and manageable within the context of a clinical trial
	Lynch [74]	Phase II	Omaveloxolone significantly improved neurological function compared to placebo and was generally safe and well tolerated	Headache, nausea, and fatigue were also more common among patients receiving omaveloxolone

Ursodiol	Peng, 2014 [75]	Phase I	Oral treatment with ursodiol prevents a toxic bile acid from causing DNA damage and NF‐κB activation in the metaplastic mucosa of patients with Barrett’s esophagus	Most are mild to moderate in severity, with a predominance of common symptoms and laboratory abnormalities that are monitorable and manageable within the context of a clinical trial
	Lakic [76]	Phase I	Reduction of liver enzymes (hepatoprotective effect), prooxidative parameters, and body mass index. Increased levels of antioxidants (SOD, GSH)	No adverse effects were reported

**Table 3:** Pharmacological activators of Nrf2—In vivo studies.

Nrf2 activator	Study (author and year)	Principal findings
Dimethyl fumarate	Lal [87]	Antiarthritic activity by enhancing the nociceptive threshold, improving arthritis scores, and reducing paw edema. Also, DMF suppressed changes in oxidative stress markers and inflammatory mediators and enhanced Nrf2 and HO‐1 levels
	McCallum [88]	DMF normalized the expression of several genes related to inflammatory and antioxidant processes in male rats with chronic unpredictable stress

Bardoxolone	Ciapala [89]	Bardoxolone methyl has shown potential to enhance opioid‐induced analgesia by acting to reduce thermal hypersensitivity in a mouse model of neuropathic pain
	Yang [80]	Significant reduction in matrix degradation, cartilage ulceration, and chondrocyte loss. In addition, significant increase in bone/trabecular volume (BV/TV) and reduction in the number of TRAP‐positive osteoclasts

Oltipraz	Zeng [90]	Oltipraz dose‐dependently inhibited joint inflammation, mechanical and heat hypersensitivities. Furthermore, it reversed gait impairment without altering locomotor activity, also reducing neutrophil infiltration in ankle joints
	Kamisako [91]	Oral administration of oltipraz suppressed the degree of inflammation and fibrosis in the liver of Nrf2‐null mice fed a high‐fat diet

Omaveloxolone	Sun [92]	The compound significantly reversed mechanical allodynia and thermal hyperalgesia in a dose‐dependent manner in a chronic constriction injury mice model
	Yang [93]	Improvements in body weight and motor function in induced motor neuron ferroptosis mice model

Ursodiol	Li [94]	Increased levels of antioxidant components (SOD, CAT, and GSH), as well as decreased of ROS and MDA (oxidative stress components)
	Li [95]	High‐dose alleviates liver inflammation by modifying gut microbiota composition and serum bile acid profiles

**Table 4:** Pharmacological activators of Nrf2—In vitro studies.

Nrf2 activator	Study (author and year)	Principal findings
Dimethyl fumarate	Cen [77]	Reduction of asthma symptoms, IgE levels and expression of inflammatory cytokines (IL‐4, IL‐13, and IL‐17). Relief of inflammation of nasal and bronchial tissues
	Mantione [78]	Reduction in ATP levels suggesting therapeutic potential in sensitizing leukemic cells to cell death

Bardoxolone	Han [79]	Nrf2 activation decreases the expression of proinflammatory cytokines such as TNF‐α and IL‐6, reducing inflammation and blocks the HIV replication
	Yang [80]	Bardoxolone inhibited ROS production and NF‐κB pathway activation, as well as inhibited osteoclast activity maintaining the bone structure

Oltipraz	Jiang [81]	The Nrf2/HO‐1 activation by oltipraz exhibited better ROS‐scavenging proficiency and greater antiapoptotic ability to protect mitochondrial membrane potential of chondrocytes
	Jie [82]	Oltipraz significantly blocked the release of inflammatory factors (TNF‐α, IL‐1β, and IL‐6) and enhancement of proliferative activity in HFLS induced by TGF‐β1

Omaveloxolone	Lin [83]	Omaveloxolone ameliorated NLRP3 inflammasome activation and mitophagy downregulation in microglia, ultimately improving cognitive impairment in the presence of alcohol
	Jiang [84]	Antioxidant, anti‐inflammatory, antiapoptotic, and anti‐extracellular matrix degradation effects through activation of the Nrf2/ARE signaling pathway and inhibition of the NF‐κB signaling pathway in chondrocytes in a ostheoarthritis model

Ursodiol	Wang [85]	Ursodiol significantly improved the level of oxidative stress and the transcription levels of inflammation and oxidative stress‐related genes in H2O2‐injured L‐02 cells
	Shimoyama [86]	Suppression of IFN‐γ production and CX3CL1 expression by ursodiol attenuated the chemotactic and adhesive abilities of liver‐infiltrating mononuclear cells, ameliorating biliary inflammation

### 3.8. Nrf2 in Neurodegeneration and Senescence

The Keap1–Nrf2 pathway plays a central role in antioxidant defense and the maintenance of redox homeostasis in the central nervous system, influencing key processes associated with brain aging and neurodegeneration. Impairments in the inducibility of Nrf2 target genes, as well as dysfunctions in Keap1‐mediated regulation, contribute to the accumulation of oxidative stress, disruption of proteostasis, and increased neuronal vulnerability to protein aggregates characteristic of disorders such as Alzheimer’s disease and tauopathies [96].

Recent studies point to a direct interconnection between Nrf2 and TAU pathology: the age‐related decline in Nrf2’s protective activity may foster aberrant TAU phosphorylation, aggregation, and neurotoxicity, whereas strategies that enhance Nrf2 signaling show potential to mitigate synaptic dysfunction and neuronal degeneration associated with TAU‐related diseases [97].

In parallel, ferroptosis, a form of iron‐dependent cell death marked by lipid peroxidation, has emerged as a relevant mechanism in neurodegeneration and is tightly regulated by Nrf2 through the control of antioxidant genes and the GPX4/selenoprotein system. Nrf2 activation promotes the expression of antioxidant components that counteract lipid peroxidation and, consequently, susceptibility to ferroptosis, positioning Nrf2 as a therapeutic intersection linking oxidative stress, iron metabolism, and neuronal loss [98, 99].

Beyond its direct effects on neurons, Nrf2 also modulates glial responses and neuroinflammation: interventions that enhance Nrf2 signaling in astrocytes have been shown to reduce the transition to neurotoxic astroglial phenotypes and to restore neuronal support functions, resulting in cognitive improvement in preclinical models of neurodegeneration. These findings underscore that Nrf2‐mediated protection operates through both cell‐autonomous (neuronal) and non–cell‐autonomous (glial) mechanisms [100].

Finally, the relationship between Nrf2 and cellular senescence extends the impact of this pathway beyond neural tissue. Nrf2 signaling, in coordination with pathways such as HIF2α, has been shown to attenuate endothelial senescence phenotypes and preserve intercellular junctions, indicating a systemic role for Nrf2 in mitigating vascular and inflammatory dysfunctions associated with aging, factors that, in turn, influence cerebral vulnerability to neurodegeneration. Thus, pharmacological or nutritional modulation of Nrf2 emerges as a promising approach to intervene in the interconnected pathological nodes linking senescence, neuroinflammation, ferroptosis, and protein aggregation [101].

## 4. Pharmacological Activators of Nrf2

### 4.1. Dimethyl Fumarate

Dimethyl fumarate (DMF) is a compound derived from fumaric acid, widely used in the treatment of autoimmune and inflammatory diseases. Originally approved for the treatment of psoriasis, DMF has gained prominence in medicine primarily due to its immunomodulatory and antioxidant properties. Its efficacy is attributed to its ability to modulate the immune system, reducing inflammation and promoting cell regeneration [102]. In recent years, the application of DMF has been expanded to the treatment of relapsing‐remitting multiple sclerosis, showing a significant impact on reducing relapse rates and disease progression [102].

A key mechanism underlying the therapeutic effects of DMF is its activation of the Nrf2 signaling cascade, leading to robust antioxidant and anti‐inflammatory responses, resulting in the induction of antioxidant enzymes, such as heme oxygenase‐1 (HO‐1), which play a crucial role in cellular defense against oxidative damage and in modulating the immune system [102] This mechanism of action is especially relevant in diseases like multiple sclerosis and rheumatoid arthritis, where oxidative stress is a major contributor to disease progression [87].

DMF has been shown to be effective in treating moderate to severe plaque psoriasis, not only reducing the severity of skin lesions but also improving the quality of life for patients. The safety of DMF was comparable to that of other established treatments, such as Fumaderm, with well‐tolerated side effects, reinforcing its suitability for clinical use [103]. Thus, the literature highlights the efficacy of DMF in cases of severe psoriasis, emphasizing its ability to maintain therapeutic results in the long term, with a continuous reduction in skin lesions and improvement in inflammatory parameters [68].

In addition to treating psoriasis, DMF has been shown to be beneficial in models of induced arthritis. Activation of the Nrf2/HO‐1 pathway by the compound resulted in significant attenuation of inflammation and alleviation of disease symptoms in a rat model of induced arthritis, suggesting therapeutic potential for the treatment of chronic inflammatory conditions [87]. Similarly, in multiple sclerosis, DMF was shown to be safe and effective in the long term, significantly reducing relapse rates and delaying disability progression in patients with the disease [67] Furthermore, Nrf2 activation by DMF attenuates periodontal inflammation and protects against tissue destruction, suggesting its potential as a therapeutic adjuvant in the treatment of periodontitis. These findings reinforce the role of DMF as a sustainable therapeutic option for managing certain diseases, such as multiple sclerosis and periodontal disease.

DMF may also be beneficial in the treatment of allergic asthma. The compound reduced allergic inflammation by enhancing the Nrf2 pathway in regulatory T cells, indicating potential use in respiratory diseases [77]. In addition to these applications described above, DMF has been explored in other emerging therapeutic areas, describing new potential applications, including its use in neurodegenerative and cardiovascular diseases [104]. These studies indicate that DMF may have a role in protecting against oxidative damage in endothelial cells, suggesting a positive impact on the prevention of age‐related diseases [105].

Collectively, these findings underscore the therapeutic versatility of DMF, whose safety and efficacy continue to support its repositioning across diverse inflammatory and oxidative‐stress–related conditions.

### 4.2. Bardoxolone Methyl

Bardoxolone methyl, a synthetic compound belonging to the triterpene class, has garnered considerable interest for its therapeutic potential in a variety of inflammatory and degenerative disorders. Originally explored as a treatment for chronic kidney disease, bardoxolone has since displayed diverse biological activities [106], encompassing anti‐inflammatory, antioxidant, and cytoprotective effects. Its multifaceted mechanism primarily involves modulating molecular pathways central to regulating oxidative stress and inflammation, thereby positioning it as a promising therapeutic agent [106].

As a potent Nrf2 activator, bardoxolone methyl promotes the upregulation of numerous antioxidant and detoxifying enzymes, such as heme oxygenase‐1 (HO‐1) and glutathione S‐transferase [106]. These actions enhance cellular resilience against oxidative stress and inflammation, safeguarding tissues from injury. Consequently, bardoxolone‐mediated Nrf2 activation has been implicated in the suppression of inflammatory pathways, marking it as a compelling therapeutic option for chronic inflammatory conditions [69].

Both preclinical and clinical investigations have yielded considerable evidence regarding bardoxolone’s effects across various medical conditions [70]. For instance, the compound has demonstrated a notable capacity to lower the urinary albumin‐to‐creatinine ratio in individuals with type 2 diabetes and stage 4 chronic kidney disease, proposing an innovative strategy for managing diabetic nephropathy [70]. Nevertheless, it is critical to acknowledge that bardoxolone methyl previously triggered safety concerns, specifically during the BEACON trial (2013) [107], where its administration was linked to an elevated risk of cardiovascular events in patients with advanced chronic kidney disease. While subsequent studies, including CARDINAL and FALCON, have reassessed its safety profile with more stringent patient oversight, its clinical application continues to warrant close examination and a thorough appraisal of its risk–benefit ratio.

Bardoxolone‐induced Nrf2 activation has also exhibited protective capabilities in additional conditions, notably osteoarthritis, by inhibiting osteoclastogenesis and shielding the extracellular matrix from degradation [80].

Furthermore, bardoxolone has demonstrated efficacy in suppressing viral replication. It can impede rabies virus infection in cells via the Nrf2 pathway, suggesting potential antiviral uses and thus broadening its therapeutic scope for infectious diseases [108]. Likewise, in HIV patients, Nrf2 activation by bardoxolone has proven effective in curbing viral replication and preventing macrophage apoptosis, which could enrich the therapeutic options against HIV, particularly in approaches aimed at safeguarding immune system cells [79].

Lastly, research into neuropathic pain using animal models has indicated that bardoxolone methyl may possess notable analgesic properties [89]. It may also enhance the effects of opioid analgesics, holding significant implications for the treatment of chronic pain in humans. The compound was observed to diminish tactile and thermal sensitivity by activating Nrf2 in a murine model of chronic constriction injury. This activation contributed to pain relief in the animals through its anti‐inflammatory and antioxidant actions [90].

While studies suggest bardoxolone exhibits a favorable pharmacological profile, its application in patients with type 2 diabetes and chronic renal dysfunction necessitates meticulous monitoring. This is because bardoxolone might impact liver enzyme activity, particularly in individuals with severe renal impairment [70].

In conclusion, bardoxolone methyl acts as a powerful Nrf2 activator, demonstrating promising therapeutic benefits across a spectrum of conditions. Its mode of action, primarily revolving around Nrf2 pathway activation, underscores its capacity to mitigate oxidative damage, alleviate inflammation, and potentially address viral infections. As ongoing clinical and preclinical research progresses, bardoxolone is anticipated to solidify its role as a versatile therapeutic agent in various medical fields.

### 4.3. Oltipraz

Oltipraz is a compound widely studied for its chemoprotective properties, particularly in relation to the activation of the transcription factor Nrf2, which has shown promising effects in several pathological conditions, including hepatic fibrosis, neuropathy, and inflammatory diseases, which reinforces the therapeutic potential of this compound [72].

When investigating the efficacy of Oltipraz in patients with hepatic fibrosis or cirrhosis, the compound was shown to reduce the progression of hepatic fibrosis, possibly through the activation of Nrf2, which attenuates oxidative stress in the liver [90]. In addition to its hepatic applications, Oltipraz has also been studied for its neurological effects, which include exerting analgesic and antidepressant effects in neuropathic pain models in mice [109], thereby modulating the activation of brain immune cells. Nrf2 activation appears to be key in this process, as it reduces inflammation and oxidative stress in the central nervous system, thereby alleviating pain and associated depressive symptoms.

Oltipraz’s ability to influence the immune and inflammatory response has also been highlighted in vitro studies. When compared with other Nrf2 activators, it demonstrated significant efficacy in reducing oxidative stress and modulating macrophage polarization [110]. These effects are considered critical for the immune response and containment of bacterial infections, suggesting a broad spectrum of therapeutic applications.

In addition, Oltipraz can protect human macrophages from cell death during *Mycobacterium tuberculosis* infections [111]. This protection is attributed to the activation of Nrf2, which enhances the resistance of cells to oxidative stress induced by infection, yielding promising results for the development of new therapeutic strategies in the treatment of tuberculosis, particularly in cases of resistance [111].

The role in regulating oxidative stress also extends to bone and joint conditions, where the compound was investigated for its ability to prevent ferroptosis [112] (a type of programmed cell death characterized by lipid peroxidation and dependent on iron) in osteoblasts induced by nanoparticles. Regulation of the Nrf2/ARE pathway may be crucial in preventing peri‐implant osteolysis, a common complication associated with orthopedic implants, as chronic inflammation around implants can create an iron‐rich environment and induce oxidative stress. Increased iron and ROS contribute to ferroptosis in cells, leading to degradation of the bone matrix and resulting in osteolysis. Furthermore, inflammation exacerbated by oral pathogens may amplify the process, further increasing the risk of bone loss around the implant [112, 113].

In the treatment of metabolic diseases, Oltipraz was responsible for attenuating the progression of steatohepatitis in Nrf2 knockout mouse models [91], suggesting that its effects may be partially independent of Nrf2 or involve other compensatory mechanisms. Regarding the anticancer potential of the compound, studies suggest that Nrf2 activation may inhibit tumor growth in glioblastoma, an aggressive type of brain cancer [114]. The main mechanisms behind this antitumor effect are the reduction of oxidative stress and modulation of inflammatory pathways by the transcription factor.

These studies highlight the adaptability of Oltipraz as an Nrf2 activator with therapeutic potential in various diseases, including hepatic and neurological pathologies, as well as inflammatory, metabolic, and oncological conditions.

### 4.4. Omaveloxolone (RTA‐408)

Omaveloxolone (RTA‐408), a synthetic compound belonging to the triterpenoid family, has drawn significant research interest due to its therapeutic potential. Its primary mechanism of action involves activating the nuclear transcription factor Nrf2, a pathway that has shown promising outcomes in a range of pathological conditions [73].

By activating Nrf2, RTA‐408 has been shown to alleviate cognitive deficits and reduce NLRP3 inflammasome activation that arises from chronic alcohol exposure [83]. This protective effect is linked to its modulation of mitophagy, the process by which damaged mitochondria are selectively removed, a mechanism vital for maintaining mitochondrial balance and preventing cellular harm from oxidative stress.

Furthermore, omaveloxolone’s ability to activate Nrf2 has demonstrated considerable neuroprotective properties in experimental models of neurodegenerative conditions. For instance, in both cellular and murine models of amyotrophic lateral sclerosis (ALS) [93], Nrf2 activation successfully suppressed motor neuron ferroptosis. This neuroprotection, observed amidst progressive neuronal degeneration, strongly indicates that modulating Nrf2 could be a viable therapeutic approach for neurodegenerative diseases [93].

Regarding the management of neuropathic pain, Nrf2 activation by omaveloxolone significantly attenuated chronic constriction injury‐induced neuropathic pain in a murine model [92]. This attenuation was achieved by stimulating PGC‐1α‐mediated mitochondrial biogenesis within the spinal cord. This finding is especially pertinent given that mitochondrial dysfunction plays a significant role in the persistence of chronic neuropathic pain [115].

Additionally, a study revealed that omaveloxolone’s inhibition of KEAP1, which negatively regulates Nrf2, could offer neuroprotection and impede the onset of epilepsy [92]. This observation underscores the compound’s therapeutic promise in pathologies where oxidative stress is a central component of disease development, like epilepsy.

Omaveloxolone’s effectiveness has also been observed in joint conditions, including osteoarthritis. An in vitro investigation demonstrated the compound’s ability to inhibit IL‐1β‐induced chondrocyte apoptosis [84] via the Nrf2/ARE and NF‐κB signaling pathways. Furthermore, in vivo studies showed that it slowed the progression of osteoarthritis in animal models. These outcomes suggest that omaveloxolone could be a viable therapeutic choice for degenerative joint diseases [84].

Beyond its anti‐inflammatory properties, omaveloxolone has also displayed positive impacts on tissue repair. RTA‐408 activation was found to enhance the regenerative capacity of wounds [116] created by punch biopsy on the backs of mice. This indicates its potential in wound healing treatments, particularly beneficial for diabetic patients whose wound healing is often impaired by heightened oxidative stress and chronic inflammation.

Collectively, these investigations highlight the extensive therapeutic promise of omaveloxolone as an Nrf2 activator, with potential uses spanning neuroprotection, wound healing, and even oncology. A deeper understanding of its precise mechanisms and the outcomes of future clinical trials will be essential in fully realizing the impact of this molecule across diverse medical fields.

### 4.5. Ursodiol (Ursodeoxycholic Acid)

Ursodiol, also known as ursodeoxycholic acid, is a naturally occurring primary bile acid found in small quantities within the human body, and notably, it was initially identified in bears [95]. This compound is extensively employed in managing various liver conditions, including primary biliary cirrhosis (PBC) and primary biliary cholangitis, owing to its well‐established cytoprotective, anti‐inflammatory, and immunomodulatory attributes. Its influence on the Nrf2 signaling pathway has particularly garnered significant attention [117].

Investigations into Ursodiol’s effects in patients with primary biliary cirrhosis [117] revealed a notable Nrf2 activation in their livers posttreatment. This suggests that the compound may aid in mitigating hepatic oxidative stress and inflammation through the Nrf2 pathway [117]. Additionally, in a murine model of acute aortic dissection [118], Ursodiol treatment significantly reduced aortic dissection formation. This positive outcome was coupled with decreased levels of ROS and enhanced Nrf2 activation, highlighting a substantial protective effect.

Within the realm of arsenic‐induced liver toxicity, Ursodiol provided substantial hepatic protection via Nrf2 pathway activation [94]. This action led to a reduction in lipid peroxidation and cellular apoptosis, thereby demonstrating its effectiveness as a hepatoprotective agent in scenarios involving toxin exposure.

Beyond its hepatoprotective actions, Ursodiol also exerts immunomodulatory effects. It can decrease T‐cell infiltration in the liver and modulate the production of IFN‐γ and CX3CL1, contributing to reduced hepatic inflammation in primary biliary cholangitis [86]. In the domain of metabolic disorders, Ursodiol treatment has improved metabolic parameters and lowered oxidative stress in patients with type 2 diabetes, further substantiating the link between Nrf2 activation and its observed antioxidant and anti‐inflammatory advantages [76].

Finally, observations indicate that high doses of Ursodiol [95] mitigated hepatic inflammation in a model of nonalcoholic steatohepatitis (NASH). This effect was linked to both the activation of Nrf2 and a beneficial remodeling of the intestinal microbiome and bile acid profile [119], thereby emphasizing the intricate cross‐talk between the hepatic and intestinal systems.

In summary, Ursodiol is a versatile compound, renowned not only for its established role in treating liver diseases but also as a potent activator of the Nrf2 pathway. Its actions extend to enhancing antioxidant defenses, curbing inflammation, and safeguarding against cellular damage across a diverse array of conditions, from aortic dissections to arsenic‐induced liver toxicity. This multifaceted functionality underscores its significant therapeutic potential in numerous pathological states.

## 5. Nonpharmacological Activators of Nrf2

### 5.1. Curcumin

Curcumin, a prominent polyphenol sourced from *Curcuma longa* (turmeric), is recognized for its notable anti‐inflammatory, antioxidant, and anticancer attributes. A key mechanism through which it exerts these diverse effects involves the activation of the Nrf2 transcription factor [120].

Research indicates that curcumin can reduce urinary albumin excretion in individuals with type II diabetes [121]. This effect is partly attributed to its capacity to enhance Nrf2 system activity and suppress inflammatory signaling pathways. Ultimately, curcumin demonstrated an improvement in renal function in diabetic patients by mitigating inflammation and oxidative stress, holding significant implications for preventing renal complications [121].

In studies where rats were pretreated with curcumin via gavage before intraperitoneal LPS injection to induce inflammation [122], modulation of Nrf2 activity was observed. This modulation subsequently attenuated “sickness behavior” and fever. Such findings suggest that curcumin possesses neuroprotective properties by regulating inflammatory responses within the brain.

In an experimental rat model of glomerular hypertension, induced by partial nephrectomy (removal of kidney tissue), curcumin was administered to assess its potential in mitigating hypertension and renal injury through Nrf2 activation [123]. Acting via Nrf2, the compound reduced oxidative stress and helped maintain the activity of crucial antioxidant enzymes such as catalase and superoxide dismutase. These outcomes suggest that the Nrf2 pathway, by upregulating antioxidant enzymes, can confer protection against hypertension and kidney damage stemming from oxidative stress [123].

A clinical trial exploring the use of oral nano‐curcumin in patients with gingivitis and mild periodontitis [124] investigated its efficacy in reducing gingival inflammation, leveraging its potent antioxidant and anti‐inflammatory properties. The compound’s activation of Nrf2 led to a decrease in inflammatory mediators and a boost in local antioxidant defense. This contributed to a reduction in the inflammatory process and alleviated gingival bleeding, a prevalent symptom in both gingivitis and mild periodontitis [124].

Concerning obesity, a study using mice fed a high‐fat diet to induce obesity examined the effects of tetrahydrocurcumin (THC) on cutaneous inflammation and oxidative stress [125]. Oral THC administration activated the Nrf2 pathway, resulting in enhanced antioxidant activity and diminished expression of inflammatory markers, including TNF‐α. Furthermore, it reduced levels of Nox2 and Nox4, known sources of ROS. These mechanisms collectively aided in mitigating oxidative stress and inflammation, conditions frequently exacerbated by obesity [125].

In cases of particle‐induced lung injury [126], curcumin plays a crucial role by alleviating damage through a dual regulation of macrophage inflammation. This involves the modulation of both the NF‐κB and Nrf2 pathways, positioning curcumin as a potential therapeutic agent for respiratory ailments stemming from environmental pollutants.

Moreover, curcumin has demonstrated effectiveness in reducing gentamicin‐induced renal and cardiac toxicity [127]. Its ability to modulate the Keap1/Nrf2, NF‐κB/iNOS, and Bcl‐2/BAX pathways underscores its extensive capacity for cellular protection against damage caused by nephrotoxic drugs, which could lead to significant clinical applications [127].

Finally, the bisdemethoxycurcumin form of curcumin has been shown to safeguard chondrocytes and lessen cartilage inflammation by modulating the NRF2/HO‐1/NLRP3 pathway [128]. These findings, coupled with research on curcumin’s application in treating traumatic brain injury [120], highlight its broad potential as a versatile therapeutic agent capable of mitigating various inflammatory and oxidative conditions via Nrf2 activation.

### 5.2. Sulforaphane

Sulforaphane, a natural compound predominantly found in cruciferous vegetables such as broccoli and cauliflower, is an isothiocyanate recognized for its potent antioxidant, anti‐inflammatory, and anticancer properties. It forms from glucoraphanin, a precursor present in these vegetables, through the enzymatic action of myrosinase [129]. Considered one of the most powerful natural Nrf2 activators, ingested sulforaphane initiates the dissociation of the KEAP1‐Nrf2 complex. This allows Nrf2 to effectively engage in cellular protection, and its activation has been linked to positive outcomes in various conditions, including cancer, cardiovascular, neurodegenerative, and inflammatory diseases [129].

Consuming sulforaphane‐rich broccoli sprouts has been correlated with decreased *Helicobacter pylori* colonization and a reduction in gastritis, as observed in both murine and human models [130]. This highlights sulforaphane’s capacity to modify the gastric environment, suggesting its effects are partially mediated by Nrf2 activation, which, in turn, fosters an antioxidant response safeguarding gastric cells.

Research also underscores sulforaphane’s significance in safeguarding against sodium dextran sulfate‐induced colitis [130]. Findings reveal that the compound’s protective actions stem not only Nrf2 activation but also its ability to modulate the intestinal microbiota. This interaction with gut bacteria appears to be critical for the full manifestation of its beneficial impacts [131].

Similarly, sulforaphane exhibits anti‐inflammatory effects, evidenced by its role in preventing LPS‐induced inflammation in goat mammary epithelial cells and in a murine model of mastitis. In these contexts, it regulates the Nrf2‐mediated autophagy pathway [132]. While Nrf2 activation mediates many of sulforaphane’s attributed effects, evidence suggests that additional mechanisms also contribute to the resolution of inflammation [132].

The compound’s efficacy was also explored for myocardial infarction treatment [133], specifically examining the targeted delivery of sulforaphane to the heart using porous magnetic nanoparticles post‐infarction. This strategy not only elevated sulforaphane concentration in cardiac tissue but also enhanced recovery, likely attributable to Nrf2 activation and the subsequent reduction of oxidative stress [133].

Furthermore, sulforaphane has been shown to alleviate psoriasis by bolstering antioxidant defenses via KEAP1‐Nrf2 pathway activation and reducing inflammatory signaling [134]. It also offers protection against various toxic substances and environmental contaminants, underscoring its potential in averting agent‐induced damage [129]. Collectively, these studies indicate that sulforaphane, through its activation of Nrf2, fulfills a multifaceted role in health promotion and disease prevention.

It is important to note, however, that not all studies have reported positive outcomes concerning Nrf2 activation by sulforaphane. For instance, oral administration of compound did not significantly impact Nrf2 expression in patients with chronic obstructive pulmonary disease (COPD) [135]. This particular study implies that sulforaphane’s efficacy might fluctuate based on the specific disease context and individual patient characteristics, emphasizing the necessity for additional research to fully elucidate the underlying mechanisms.

### 5.3. Resveratrol

Resveratrol, a natural polyphenol prevalent in various plants such as grapes, berries, and peanuts, has attracted considerable scientific interest. Its prominence stems from its powerful antioxidant, anti‐inflammatory, and antitumor properties, largely attributed to its interaction with multiple cellular pathways. These interactions foster cardiovascular health, offer protection against cancer, and may decelerate cellular aging [136]. A key aspect of resveratrol’s protective actions is its capacity to activate the transcription factor Nrf2, which serves as a central mechanism for enhancing cellular antioxidant capacity and mitigating the effects of inflammatory and degenerative conditions [136].

Resveratrol demonstrates a significant protective impact on the kidneys of rats afflicted with diabetic nephropathy [137]. It achieves this by reducing endoplasmic reticulum stress, a pivotal factor in the development of disease. This beneficial effect is partly facilitated by Nrf2 activation, which, in turn, boosts the expression of antioxidant enzymes, thereby shielding kidney cells from oxidative and inflammatory damage.

The capacity of resveratrol to influence multiple signaling pathways is further underscored by its role in attenuating radiation enteritis, an inflammatory intestinal condition often arising post‐radiotherapy [138]. The protection afforded by resveratrol encompassed the modulation of the SIRT1/FOXO3a and PI3K/AKT pathways, alongside Nrf2 activation. These combined actions contributed to diminishing inflammation and oxidative stress within intestinal tissues [138].

Resveratrol’s protective capabilities are also exemplified by its ability to shield retinal ganglion cells from damage in diabetic models [139], primarily via the activation of the Nrf2 signaling pathway. Nrf2 activation spurred an upregulation of antioxidant genes, which consequently reduced cellular apoptosis and helped preserve visual function. This suggests that resveratrol could emerge as a promising therapeutic candidate for diabetic retinopathy [139].

Finally, this polyphenol has demonstrated antitumor properties by interfering with the mechanisms of *Helicobacter pylori* [140], a bacterium linked to gastric cancer. Beyond its antimicrobial effects, resveratrol also activated the Nrf2 pathway, which contributed to reduced oxidative stress and inflammation in the gastric milieu, potentially inhibiting cancer progression [140]. In a similar vein, resveratrol also suppresses the proliferation of prostate cancer cells. Here, Nrf2 activation and HO‐1 induction were pivotal mechanisms in this inhibition, suggesting that the compound can rebalance oxidative stress within cancer cells, fostering cell death and curbing tumor advancement [141]. These collective findings underscore resveratrol’s significant potential as a compound capable of multifaceted actions to promote health and prevent disease.

### 5.4. Challenges in the Bioavailability of Natural Nrf2 Activators

Although natural Nrf2 activators such as curcumin, sulforaphane, and resveratrol exhibit potent antioxidant and anti‐inflammatory actions in experimental models, their clinical translation remains significantly hindered by poor bioavailability. This discrepancy between preclinical efficacy and human outcomes arises from multiple pharmacokinetic constraints that limit their absorption, systemic distribution, metabolic stability, and ability to reach therapeutic concentrations in target tissues.

Curcumin exemplifies these limitations. Despite its wide‐ranging biological effects, it possesses extremely low aqueous solubility, undergoes rapid intestinal and hepatic conjugation, and is quickly eliminated from the circulation. Even high oral doses result in minimal plasma levels, predominantly in metabolized forms with substantially reduced biological activity. These factors restrict the compound’s capacity to achieve sustained Nrf2 activation in vivo, helping explain the modest or inconsistent clinical results reported for conventional formulations [142, 143].

Sulforaphane, although one of the most potent natural Nrf2 activators, faces a distinct set of barriers. Its generation from glucoraphanin requires enzyme myrosinase, which is easily inactivated by cooking and processing, and its conversion is heavily dependent on gut microbiota composition. As a result, individuals consuming identical quantities of cruciferous vegetables may exhibit up to a fivefold variation in circulating sulforaphane levels. Additionally, sulforaphane is chemically unstable and undergoes rapid conjugation with glutathione, leading to swift renal excretion and limiting its tissue residence time [144].

Resveratrol, widely studied for its cardioprotective and antitumor properties, also exhibits notoriously low oral bioavailability. Although it is readily absorbed, it undergoes extensive first‐pass metabolism to glucuronide and sulfate conjugates, resulting in minimal free resveratrol reaching systemic circulation. Its brief half‐life further complicates efforts to maintain sufficient concentrations to drive robust Nrf2 activation, contributing to variability in therapeutic outcomes across preclinical and clinical studies [145, 146].

Given these challenges, there is growing recognition that enhancing bioavailability is essential for unlocking the full therapeutic potential of natural Nrf2 activators. Innovative strategies, including nanoencapsulation, liposomal delivery, solid lipid nanoparticles, phospholipid complexes, and the use of absorption enhancers such as piperine, have shown promise in improving solubility, protecting compounds from premature metabolism, and prolonging systemic exposure. Food‐based delivery systems, co‐ingestion with dietary fats, and microbiota‐targeted strategies have also emerged as viable methods to optimize absorption and metabolic conversion [147, 148].

Together, these advances underscore that the future clinical relevance of natural Nrf2 activators depends not only on their inherent biochemical properties but also on the development of delivery platforms capable of overcoming their pharmacokinetic limitations. Improving bioavailability is therefore a critical step toward ensuring consistent Nrf2 activation, enhancing therapeutic efficacy, and facilitating their integration into evidence‐based clinical practice.

## 6. Clinical Safety of Nrf2 Activators

This review highlights recent advances that elucidate the connection between Nrf2 modulation and the progression of several highly prevalent diseases (Table 1). It also discusses the therapeutic potential of Nrf2 activators, drawing upon evidence from both preclinical and clinical studies (Table 2).

Among the compounds evaluated, dimethyl fumarate stands out as the only Nrf2 activator currently approved by the FDA, indicated for the treatment of psoriasis and multiple sclerosis [149, 150]. Furthermore, it has demonstrated efficacy and safety in the management of other inflammatory conditions, such as rheumatoid arthritis and asthma. Other activators reviewed have also shown favorable safety profiles, typically characterized by expected or non‐serious adverse effects. (Tables 2–4).

## 7. Conclusion

Activating the transcription factor Nrf2 is a critical cellular defense mechanism against oxidative stress, functioning as a central regulator for genes involved in antioxidant and cytoprotective responses. Across this review, we have shown how numerous natural and synthetic compounds positively influence the Nrf2 pathway, helping protect cells from chemical, inflammatory, and environmental damage.

However, it is vital to acknowledge that excessive or dysregulated Nrf2 activation can lead to adverse effects. This is especially true under conditions of chronic exposure or in certain diseases like cancer, where continuous activation might inadvertently promote the survival and proliferation of malignant cells.

Consequently, gaining a more comprehensive understanding of the Nrf2 pathway, encompassing its modulators and their associated pathophysiological consequences, is crucial for developing safer and more effective therapeutic strategies. Nrf2’s dual role highlights the necessity for personalized approaches that account for the specific cellular context, as well as the intensity and duration of activation, to maximize its protective advantages while mitigating potential risks.

Looking forward, future research should prioritize translational strategies that address current limitations in bioavailability, optimal dosing, and systemic safety. Innovations such as nanotechnology‐based delivery systems and prodrug formulations could enhance tissue‐specific Nrf2 activation, effectively minimizing off‐target effects. Simultaneously, developing robust in vivo biomarkers to monitor Nrf2 activation and downstream gene expression would significantly improve therapeutic oversight. Ultimately, personalized interventions, tailored by polymorphisms in NRF2, KEAP1, or related regulatory genes, might enable precise modulation of this pathway, thereby optimizing both efficacy and safety across diverse patient demographics.

## Conflicts of Interest

The authors declare no conflicts of interest.

## Funding

No funding was received for this manuscript.

## Data Availability

Data sharing is not applicable to this article as no datasets were generated or analyzed during the current study.
